# 5-Fluorouracil upregulates cell surface B7-H1 (PD-L1) expression in gastrointestinal cancers

**DOI:** 10.1186/s40425-016-0163-8

**Published:** 2016-10-18

**Authors:** Lauren Van Der Kraak, Gaurav Goel, Krishnaveni Ramanan, Christof Kaltenmeier, Lin Zhang, Daniel P. Normolle, Gordon J. Freeman, Daolin Tang, Katie S. Nason, Jon M. Davison, James D. Luketich, Rajeev Dhupar, Michael T. Lotze

**Affiliations:** 1 Department of Cardiothoracic Surgery, University of Pittsburgh, Pittsburgh, PA USA; 2 Department of Medicine, Division of Hematology-Oncology, University of Pittsburgh, Pittsburgh, PA USA; 3 Current address: Division of Medical Oncology, University of Kentucky Markey Cancer Center, Lexington, KY USA; 4 Department of Surgery, University of Pittsburgh, Pittsburgh, PA USA; 5 Department of Pharmacology & Chemical Biology, University of Pittsburgh Cancer Institute, University of Pittsburgh School of Medicine, Pittsburgh, PA USA; 6 Department of Biostatistics, University of Pittsburgh, Pittsburgh, PA USA; 7 Department of Medical Oncology, Dana-Farber Cancer Institute, Harvard Medical School, Boston, MA USA; 8 Department of Pathology, University of Pittsburgh, Pittsburgh, PA USA; 9 Department of Immunology, University of Pittsburgh, Pittsburgh, PA USA; 10 Department of Bioengineering, University of Pittsburgh, Pittsburgh, PA USA

**Keywords:** 5-Fluorouracil, B7-H1, PD-L1, PD-1, Digestive cancers, Checkpoint blockade, Immunotherapy

## Abstract

**Background:**

Resistance to chemotherapy is a major obstacle in the effective treatment of cancer patients. B7-homolog 1, also known as programmed death ligand-1 (PD-L1), is an immunoregulatory protein that is overexpressed in several human cancers. Interaction of B7-H1 with programmed death 1 (PD-1) prevents T-cell activation and proliferation, sequestering the T-cell receptor from the cell membrane, inducing T-cell apoptosis, thereby leading to cancer immunoresistance. B7-H1 upregulation contributes to chemoresistance in several types of cancer, but little is known with respect to changes associated with 5-fluorouracil (5-FU) or gastrointestinal cancers.

**Methods:**

HCT 116 p53^+/+^, HCT 116 p53^−/−^ colorectal cancer (CRC) and OE33 esophageal adenocarcinoma (EAC) cells were treated with increasing doses of 5-FU (0.5 uM, 5 uM, 50 uM, 500 uM) or interferon gamma (IFN-γ, 10 ng/mL) in culture for 24 h and B7-H1 expression was quantified using flow cytometry and western blot analysis. We also evaluated B7-H1 expression, by immunohistochemistry, in tissue collected prior to and following neoadjuvant therapy in 10 EAC patients.

**Results:**

B7-H1 expression in human HCT 116 p53^+/+^ and HCT 116 p53^−/−^ CRC cells lines, while low at baseline, can be induced by treatment with 5-FU. OE33 baseline B7-H1 expression exceeded CRC cell maximal expression and could be further increased in a dose dependent manner following 5-FU treatment in the absence of immune cells. We further demonstrate tumor B7-H1 expression in esophageal adenocarcinoma patient-derived pre-treatment biopsies. While B7-H1 expression was not enhanced in post-treatment esophagectomy specimens, this may be due to the limits of immunohistochemical quantification.

**Conclusions:**

B7-H1/PD-L1 expression can be increased following treatment with 5-FU in gastrointestinal cancer cell lines, suggesting alternative mechanisms to classic immune-mediated upregulation. This suggests that combining 5-FU treatment with PD-1/B7-H1 blockade may improve treatment in patients with gastrointestinal adenocarcinoma.

## Background

5-Fluorouracil (5-FU), a uracil mimetic, is a chemotherapeutic drug commonly used to treat patients with advanced anal, colorectal (CRC), stomach, breast, esophageal and head/neck cancers [1–4]. It induces cell death through the inhibition of thymidylate synthase and through its misincorporation into newly synthesized DNA and RNA [4]. 5-FU exerts the most robust response in combination with irinotecan and oxaliplatin in CRC, with response rates of 40-50 % [4]. Both innate and acquired chemoresistance continue to be important obstacles in the effective treatment of patients using 5-FU.

In recent years, there has been increasing evidence to support a role for the immune system in effective 5-FU treatment and the development of chemoresistance [5]. These include induction of Heat Shock Protein 70 on tumor cells following treatment leading to enhanced tumor uptake by dendritic cells. Subsequent IL-12 secretion and enhanced antigen presentation and induced expression of Intercellular Adhesion Molecule 1 and Fas ligand can result in tumor cell elimination by T-cells [6, 7]. Reduction in the frequency of circulating and tumor-infiltrating myeloid-derived suppressor cells (MDSCs) via induction of 5-FU-induced apoptotic cell death may itself promote changes in the expression of B7-H1 [5–8].

B7-homolog 1 (B7-H1), also known as programmed death ligand-1 (PD-L1), is an immunoregulatory protein that belongs to the B7 family of T-cell co-regulatory molecules [9]. It is one of two ligands for the PD-1 receptor (CD279), a costimulatory molecule expressed on the surface of T-cells [10]. Interaction of B7-H1 with PD-1 prevents T-cell activation and proliferation, thus inducing T-cell apoptosis, leading to cancer immunoresistance [11]. B7-H1 is overexpressed in solid cancers, including breast, colon, esophageal, gastric, lung, ovarian and pancreatic cancers, and is often categorized as a poor prognostic factor, although occasionally it has been shown as a favorable factor [12]. Interestingly B7-H1 expression can be induced in cells and tissue following treatment with chemotherapeutic agents. For example, *McDaniel* et al. demonstrated increased B7-H1 in urothelial carcinoma tumor cores following treatment with cisplatin/carboplatin [13]. Paclitaxel induces B7-H1 expression in the human colon cancer cell-line SW480 and the hepatocellular carcinoma cell-line HepG2 via the mitogen-activated protein kinase pathway [14]. However, little is known about the effects of 5-FU treatment on B7-H1 expression in digestive cancers, although 5-FU treatment upregulates B7-H1 in MDA-MB 408 and 435 breast cancer cell lines, but not MCF-7 cells [15].

Herein, we investigate B7-H1 expression following treatment with 5-FU in several gastrointestinal cancer cell lines. Mutations in the p53 tumor suppressor have been associated with both poor responsiveness to 5-FU and microRNA-34 upregulation of B7-H1 [16–19]. Therefore, we investigated B7-H1 expression following 5-FU treatment in both HCT 116 p53^+/+^ and HCT 116 p53^−/−^ CRC cells. We also investigated B7-H1 expression in OE33 Barrett’s-derived esophageal adenocarcinoma cells, since B7-H1 expression has been found in patients with advanced Barrett’s carcinoma, but the influence of chemotherapy on B7-H1 is not known [20].

## Methods

### Cell culture

Human colorectal cancer cell lines (HCT 116 p53 ^+/+^, HCT 116 p53 ^−/−^, HT29 and SW480) were obtained from Dr. Edward Chu and Dr. Lin Zhang (University of Pittsburgh Medical Center) and confirmed to be mycoplasma negative using the MycoAlert^TM^ mycoplasma detection kit (Lonza Group Ltd, Allendale, NJ). OE33, esophageal adenocarcinoma cells from a patient with Barrett’s esophagus were purchased from Sigma Aldrich (St. Louis, MO). All cells were grown in RPMI 1640 plus 2.05 mM glutamine media that had been supplemented with 1× penicillin-streptomycin and 10 % fetal bovine serum, and were maintained in an incubator at 37 °C in 5 % CO_2_.

### 5-FU and IFN gamma treatment

On the day of treatment, cells were trypsinized and seeded into 6-well plates. The cell volume was calculated to correspond to 75–85 % confluency in the untreated wells at time of harvest. Six hours post-plating, cells were treated with plain media, 5-fluorouracil (5-FU; APP Pharmaceuticals LLC, Schaumberg, IL) or interferon gamma (IFN- γ; Gemini Bio, West Sacramento, CA) according to the doses in the results section of this paper. Cells were harvested 24 h after treatment initiation.

### Western blot analysis

Twenty-fours hours after treatment initiation, the media was removed and cells were washed with ice-cold phosphate-buffered saline (PBS). The cells were trypsinized, collected and washed again with PBS to remove residual trypsin. The cells were lysed in 25 ul of Cell Lysis Buffer (BD Biosciences, San Jose, CA) containing Halt Protease Inhibitor Cocktail (Thermo Scientific, Rockford, IL). The lysates were centrifuged and the supernatant was collected and stored at −80 °C. Protein quantification was done using the Pierce BCA Protein Assay (Thermo Scientific, Rockfold, IL). Forty micrograms of protein per treatment condition was loaded onto 4–12 % Bis-Tris gradient gels and transferred onto nitrocellulose membranes (BioRad, Hercules, CA). The membranes were blocked with 5 % non-fat dry milk in PBS-Tween (PBS-T, 0.2 % Tween) and incubated overnight at 4 °C with mouse monoclonal purified anti-human B7-H1 antibody (clone 9A11, diluted 1:100 CRC cells, 1:250 OE33). The 9A11 antibody was a generous gift from Dr. Gordon Freeman (Dana Farber Cancer Institute, Boston, MA). All dilutions were made with 5 % non-fat dry milk in PBS-T. After washing with PBS-T, the membranes were incubated with horseradish peroxidase (HRP)-conjugated mouse secondary antibody (1:1000) at room temperature. The membranes were washed and the bands were visualized using Clarity Western ECL Substrate (BioRad, Hercules, CA) and Hyclone Film (Thermo Scientific, Rockford, IL). Membrane were subsequently stripped and re-probed for actin (1:1000–1:5000 dilutions, Sigma Aldrich, St. Louis, MO).

Western blots were quantified using ImageJ software Version 1.50 g. The quantified data are presented as a ratio of PD-L1 Band Intensity (as calculated by ImageJ) divided by Actin Band Intensity.

### Flow cytometry

Twenty-fours hours after treatment initiation, the media was removed and cells washed with ice-cold phosphate buffered saline (PBS). The cells were trypsinized, collected and washed with PBS to remove residual trypsin. Cells were incubated with PE-anti human CD274 (B7-H1, clone MIH1) or isotype control antibodies (BD Biosciences, San Jose, CA) for 30 min at 4 °C. Cells were then washed with PBS before being fixed in 1 % paraformaldehyde. B7-H1 surface expression was read using a BD Accuri C6 flow cytometer (BD Biosciences, San Jose, CA). The mean fluoroscent intensity of the isotype control from each experiment was subtracted from the respective samples before plotting the data. If treated samples had lower values than the isotype control they were recorded as zero.

### PD-L1 expression in patient derived samples

Ten matched pre-neoadjuvant (cisplatin, 5-FU and radiation) and post-esophagectomy samples were provided by KSN, JMD and JDL. Samples were paraffin-embedded, cut and stained for B7-H1 using immunohistochemistry according to published procedures [21]. Samples were scored in a blinded fashion by LVDK and images captured using the EVOS FL Auto Microscope (Life Technologies, Waltham, MA). Samples were considered positive if 1 % of the tumor cells showed staining for B7-H1.

### Statistics

All data are presented as mean +/− the standard error of the mean unless otherwise noted. Continuous variables were compared between experimental arms using Student’s *t*-test or ANOVA. Results were considered statistically significant if *p* < 0.05. When results from multiple independent experiments are combined, mixed effects ANOVA is used to account for between-experiment variation. If an ANOVA is considered statistically significant, the p-values of post-hoc pairwise comparisons were adjusted using Westfall's method [22]. Statistical analyses were performed using GraphPad Prism and R.

## Results

### 5-FU induces B7-H1 surface expression in colorectal cancer cell lines

**Fig. 1:**
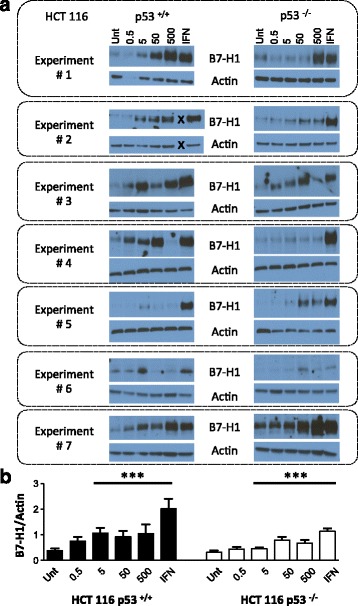
5-FU treatment promotes B7-H1 protein expression in whole cell lysates from HCT 116 WT ^+/+^ and HCT 116 p53 ^−/−^ colon cancer cell lines. **a** Western blots from seven independent experiments measuring B7-H1 and actin expression in HCT 116 p53^+/+^ and HCT 116 p53^−/−^ cells. The “X” in experiment # 2 indicates a skipped lane when loading the samples. Unt = untreated control, 0.5, 5, 50 and 500 = 5FU doses in uM, IFN = 10 ng/ul of interferon gamma. **b** Quantification of B7-H1 expression using ImageJ. Data is represented as mean B7-H1 band intensity/mean actin band intensity +/− SEM. *** *p* < 0.001 relative to the untreated control

**Fig. 2:**
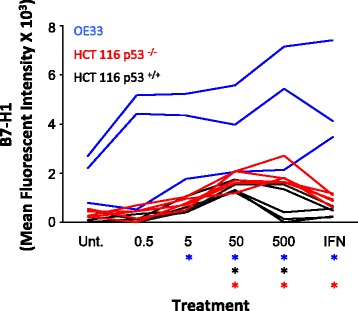
5-FU treatment promotes B7-H1 protein surface expression in HCT 116 p53^+/+^, HCT 116 p53 ^−/−^ and OE33 cells as measured using flow cytometery. Surface expression of B7-H1 expressed as mean fluorescent intensity as measured using flow. The isotype control was subtracted from each individual experiment and values measuring less than the isotype were assigned an absolute value of zero. Each line represents a single experiment. * *p* < 0.02 relative to the untreated control for that cell line

We examined B7-H1 expression in HCT 116 p53^+/+^ and HCT 116 p53 ^−/−^ cells following 24-h treatment with 5-FU (0.5 uM, 5 uM, 50 uM, 500 uM) by Western blot analysis (Fig. 1 a and b). Interferon (IFN)-ɣ is a T_H_1 cytokine produced and secreted by natural killer and T cells within the tumor microenvironment. IFN-ɣ can induce B7-H1 expression in several histologic types of tumors and in tumor cells in culture and therefore we utilized treatment with 10 ng/ul of IFN-ɣ as a positive control for B7-H1 induction [23, 24]. B7-H1 expression was barely detectable at baseline with IFN-ɣ eliciting the highest B7-H1 protein expression (*p* < 0.001 to untreated). Overall levels of B7-H1 induction were low, as Western analysis required at least 20 min to induce detectable protein expression. Treatment with doses of 5 uM or higher 5-FU induced B7-H1 expression (*p* < 0.001) under our experimental conditions in both HCT 116 p53^+/+^ and HCT 116 p53 ^−/−^ cell lines. While the HCT 116 p53^+/+^ cells had higher average B7-H1 expression (*p* = 0.013) than the HCT 116 p53^−/−^ cells, there was no significant interaction between treatment and p53 status, indicative of a similar trend in induction within both cell lines. This B7-H1 increase following 5-FU was confirmed at the 50 uM and 500 uM 5-FU doses (*p* < 0.02) for both cells lines and for IFN- ɣ in the HCT 116 p53^−/−^ cells using flow cytometry (Fig. 2).

### Colon cancer cells exhibit limited B7-H1 surface expression at baseline

**Fig. 3:**
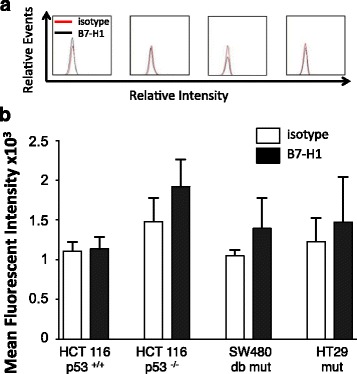
Colorectal cancer cell lines express low surface B7-H1 at baseline. **a** Representative histograms and **b** quantification of mean fluorescent intensity (MFI) for baseline B7-H1 surface expression compared to the isotype control in HCT116 p53^+/+^, HCT116 p53^−/−^, SW480 and HT29 colorectal cancer cell lines (*n* = 3). The SW480 cells carry R273H and P309S homozygous mutations at p53 and the HT29 have a homozygous R273H mutation

To determine if low baseline B7-H1 expression was indicative of other CRC cell lines we analyzed B7-H1 surface expression in HCT116 p53^+/+^, HCT116 p53 ^−/−^, SW480 and HT29 cells using flow cytometry (Fig. 3). The B7-H1 histograms remained superimposed on the isotype control staining and there were non-significant differences in mean fluorescent intensity (MFI) between the isotype control and B7-H1 samples suggestive of non-detectable B7-H1 expression in untreated CRC cells. Western analysis did not show detectable bands in these CRC cell lines (data not shown).

### 5-FU induces B7-H1 surface expression in OE33 Barrett’s adenocarcinoma cells

**Fig. 4:**
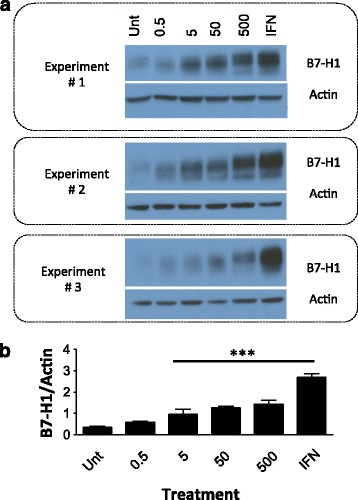
5-FU treatment promotes B7-H1 protein expression in a dose dependent manner in OE33 esophageal adenocarcinoma cells. **a** Western blots from three independent experiments measuring B7-H1 and actin expression. Unt = untreated control, 0.5, 5, 50 and 500 = doses in uM of 5FU, IFN = 10 ng/ul of interferon gamma. **b** Quantification of B7-H1 expression using ImageJ. Data is represented as mean PD-L1 band intensity/mean actin band intensity +/− SEM. *** *p* < 0.001 relative to the untreated control

To assess if B7-H1 expression was important in other gastrointestinal cancers, we examined expression of B7-H1 in esophageal adenocarcinoma OE33 cells. These cells are derived from a patient with Barrett’s adenocarcinoma, which is associated with intestinal metaplasia and aggressive malignancy. OE33 cells had higher baseline expression (untreated) relative to HCT 116 p53 ^+/+^ and HCT 116 p53 ^−/−^ cells. We showed by both Western Blot (Fig. 4) and flow cytometry (Fig. 2) that treatment with 5-FU (5 uM or higher) and IFN- ɣ induces B7-H1 expression in OE33 cells. It is important to note that with similar exposure times no evidence of B7-H1 was seen in the CRC treated cells, indicative of higher overall expression in the OE33 cells. B7-H1 expression was dose dependent in our Western analysis, with evidence of two distinct bands, with the lower band rarely seen in the CRC cell lines (Fig. 1 a). The lower band may reference a second PD-L1 isoform identified by *He* et al., but additional analysis is necessary [25]. Overall, the OE33 cells had a significant induction of B7-H1 following 5-FU treatment that was significantly higher than our CRC cell lines.

### B7-H1 protein is detectable by immunohistochemistry in esophageal adenocarcinoma biopsies

**Fig. 5:**
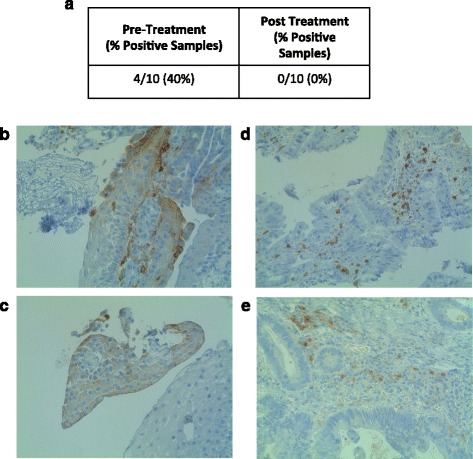
B7-H1 is not upregulated following treatment with neoadjuvant therapy in esophageal adenocarcinoma tissue samples. **a** Percent positivity of patient samples comparing pre-neoadjuvant (cisplatin, 5-FU and radiation) therapy biospies to matched post-neoadjuvant esophagectomys (cisplatin, 5-FU and radiation). **b** and **c **B7-H1 expression in two representative pre-treatment specimens using immunohistochemistry (40 ×). **d** and **e** Representative images showing B7-H1 expression on immune cells, but not on tumor cells (40 ×)

We have shown that B7-H1 expression can be induced in a 5-FU dose dependent manner in OE33 cells. To determine if a similar increase could be observed in esophageal adenocarcinoma patients treated with 5-FU containing regimes we compared PD-L1 expression in 10 matched pre- and post-treatment (5-FU, cisplatin and radiation therapy) tissue samples using immunohistochemistry. All samples showed positive scoring for B7-H1 expression on immune cells (CD68^+^ macrophages, CD3^+^ T-cells), with no obvious correlations with respect to extent of infiltrate between the pre and post treatment specimens (Figs. 5 c and d). We observed positive B7-H1 scoring in 40 % of the pre-treatment biopsies (Fig. 5 a–c). Contrary to our in vitro studies, enhanced B7-H1 expression was not observed on tumor cells in the post-treatment samples (Fig. 5 a). This may be due to the staining antibody employed and the noted difficulties in immune-histochemical consistent identification of this moiety.

## Discussion

B7-H1 is a negative co-stimulatory molecule that is expressed in many cancers, whose expression plays a pivotal role in the ability of tumor cells to evade the host response [11]. Since a few studies have also demonstrated increased B7-H1 expression following treatment with chemotherapeutic agents (since submission of this manuscript), we investigated if 5-FU could have a similar effect in gastrointestinal cancers [13–15]. We treated HCT 116 p53^+/+^ and HCT 116 p53^−/−^ CRC cells lines and OE33 Barrett’s carcinoma cells with 5-FU and studied B7-H1 expression using flow cytometry and western blot analysis.

Consistent with previous studies, we have shown that IFN-γ can induce B7-H1 protein expression [23, 24]. We also demonstrate that treatment with 5 uM or higher 5-FU can induce B7-H1 upregulation in HCT 116 cells as detected by western blot analysis, regardless of *p53* mutational status. B7-H1 surface expression, detected by flow cytometry, was observed in HCT cells treated with 50–500 uM 5-FU. Differences between western and flow analysis may reflect differences in surface vs intracellular expression or may be influenced by dead or dying cells, which can be excluded from analysis using flow. HCT 116 cells have functional *MSH2*, but non-functional *MSH1* and are therefore classified as a microsatellite instable cell line (MSI) [26, 27]. MSI tumors have been shown to upregulate multiple immune checkpoints including PD-1 and B7-H1, with microsatellite stable tumors being less responsive and therefore our HCT 116 cells could be expected to be ideal for studying B7-H1 induction [28, 29]. Recently, a phase II trial demonstrated clinical benefit of immune checkpoint blockade with an anti-PD-1 antibody (pembrolizumab) in MSI-hi CRC patients [30]. We demonstrate that baseline B7-H1 is low in SW480 and HT29 CRC cells, consistent with other studies demonstrating low B7-H1 expression on tumors at baseline [11]. Combining 5-FU-based therapy with anti PD-1/B7-H1 pathway inhibitors might be a potential strategy to overcome 5-FU-induced immunoresistance, and thereby improve the clinical outcomes of CRC patients. Further preclinical and clinical studies are warranted to formally test this hypothesis and determine if the 5-FU levels of induction correspond to therapeutically targetable levels.

B7-H1 expression was also induced in a dose dependent manner in OE33 EAC cells and to a significantly higher extent than the HCT 116 cells. Interestingly, we saw more than one band for B7-H1 in our OE33 cell western blot analysis. Additional testing is necessary to determine if these bands correspond to different B7-H1 isoforms or variations in the glycosylation pattern of the B7-H1 protein [25, 31, 32].

To test for 5-FU upregulation following neoadjuvant treatment in EAC patient samples we obtained matched pre-neoadjuvant (cisplatin, 5-FU, radiation) and post-neoadjuvant samples from 10 EAC patients. We detected positive B7-H1 staining in 40 % of pre-treatment patient samples, which was slightly higher than observed previously [12, 21]. However, this was significantly lower than *Loos* et al., who showed 73 % B7-H1 staining in Barrett’s-associated EAC, which has higher B7-H1 expression than non-Barrett’s EAC [20]. We did not detect any B7-H1 staining in our post-treatment samples. There are several possible explanations for these observations 1) B7-H1 tumor expression is heterogeneous and it is not known if our biopsy and esophagectomy samples correspond to identical tumors/regions of tumor [31]. 2) B7-H1 expression has also been demonstrated to occur primarily along the invasive front of the tumor and many post-treatment samples lacked a clearly defined regions of tumor [33]. 3) It is also not clear if B7-H1 expression is upregulated following removal of the initial 5-FU stimuli in our cell lines. Therefore, it is plausible that B7-H1 expression is transient in patients undergoing neoadjuvant therapy and therefore obtaining esophageal biopsies mid-treatment may better reflect the conditions necessary to detect B7-H1 upregulation in EAC patients. 4) B7-H1 expression is associated more strongly with Barrett’s associated EAC and the Barrett’s status of our samples is unknown. Testing of additional EAC samples from patients with advanced metastatic disease will be necessary and hopefully will demonstrate similar results to a recent study that showed increased B7-H1 expression following chemotherapy and radiation therapy for esophageal squamous cell carcinoma [34]. We also wish to the assess expression of the second PD-1 ligand, B7-DC, in these same cells and samples. *Derks* et al. have shown high levels of B7-DC in OE33 and EAC patient samples at baseline, however it is not known if chemotherapy can also alter B7-DC expression [21].

## Conclusions

B7-H1 overexpression is an important mechanism by which tumor cells can escape host-T cell immunity. We have demonstrated that while B7-H1 expression is low at baseline its expression can be induced in HCT 116 p53^+/+^, HCT 116 p53^−/−^ and OE33 cells following treatment with 5-FU, the chemotherapy of choice for both advanced CRC and EAC. We hypothesize that this increase in B7-H1 following chemotherapeutic treatment may contribute in part to the chemoresistance that develops following 5-FU treatment. While we did not find a corresponding increase in post-neoadjuvant treated esophageal adenocarcinoma tissue samples, we recognize that we had a limited number of samples and that 5-FU induced B7-H1 could be transient since the underlying mechanisms of B7-H1 upregulation remains unknown. We postulate that counteracting the immunosuppressive cofactor B7-H1 using PD-1/B7-H1 blockade might enhance the anti-cancer effects of 5-FU in the management of gastrointestinal cancers. Future experiments will therefore, involve co-culture of 5-FU treated colon cancer cells with PD-1+/CD8+ T-cells to evaluate T-cell apoptosis and will examine if anti-PD-1/B7-H1 blockade inhibits this process. This would further help establish any potential synergism between 5-FU-based chemotherapy and PD-1/B7-H1 blockade in the management of patients with gastrointestinal cancers.

## Abbreviations

5-FU: 5-Fluorouracil
B7-H1: B7 Homolog-1
B7-DC: B7 Homolog-DC
CRC: Colorectal cancer
EAC: Esophageal adenocarcinoma
IFN- γ: Interferon gamma
IL: Interleukin
MDSC: Myeloid derived suppressor cells
MSH: DNA mismatch repair protein
MSI: Microsatellite instable
PBS: Phosphate-buffered saline
PBS-T: Phosphate-buffered saline tween
PD-1: Programmed death 1
PD-L1: Programmed death ligand 1
SEM: Standard error of the mean
UPCI: University of Pittsburgh Cancer Institute
